# Dynamics of Dissolution, Killing, and Inhibition of Dental Plaque Biofilm

**DOI:** 10.3389/fmicb.2020.00964

**Published:** 2020-05-20

**Authors:** Zhejun Wang, Ya Shen, Markus Haapasalo

**Affiliations:** Division of Endodontics, Department of Oral Biological and Medical Sciences, Faculty of Dentistry, The University of British Columbia, Vancouver, BC, Canada

**Keywords:** biofilm dissolution, confocal laser scanning microscopy, chlorhexidine, extracellular polymeric substance, live-cell imaging, sodium hypochlorite

## Abstract

The present study aims to establish a standardized model that makes it possible to evaluate the dynamic dissolution of biofilm, killing of biofilm microbes and inhibition of growth of biofilm by disinfecting solutions. Biofilm was grown from dental plaque bacteria on collagen-coated hydroxyapatite (HA) disks for 3 days or 3 weeks under anaerobic conditions. Biofilms were stained with the LIVE/DEAD viability stain and subjected to sterile water, 2% sodium hypochlorite (NaOCl), 6% NaOCl, or 2% chlorhexidine (CHX) for 32 min. Dynamic change in fluorescence on bacterial cells and extracellular polymeric substance (EPS) during the exposure was analyzed using Alexa Fluor 647-labeled dextran conjugate and a live-cell imaging confocal laser scanning microscopy (LC-CLSM). The biofilm structures after treatments were visualized by scanning electron microscopy (SEM). The treated biofilms on HA disks were collected and subjected to colony forming unit (CFU) counting. Another set of sterile HA disks were coated with CHX prior to the monitoring of plaque biofilm growth for 12 h. The LC-CLSM results showed that NaOCl dissolved biofilm effectively, more so at a higher concentration and longer exposure time. Six percent NaOCl was the most effective at dissolving and killing bacteria (e.g., 99% bacterial reduction in 3-day-old biofilm and 95% bacterial reduction in 3-week-old biofilm in 32 min) followed by 2% NaOCl and CHX. Sodium hypochlorite dissolved over 99.9% of the EPS whereas CHX only slightly reduced the EPS biovolume in 32 min. CFU results indicated that the dispersed biofilm bacteria are more resistant than planktonic bacteria to disinfectants. SEM showed the disruption of biofilm after exposures to CHX and NaOCl. The use of 2% CHX and sterile water did not result in biofilm dissolution. However, prior exposure of the HA disks to 2 and 0.2% CHX for 3 min prevented biofilm from growing on the HA disk surfaces for at least 12 h. This new platform has the potential to aid in a better understanding of the antibiofilm properties of oral disinfectants.

## Introduction

Bacteria organized in multicellular biofilm communities pose a considerable clinical challenge because they are capable of causing oral diseases (Leung et al., 2005). One of the most complex biofilm systems in nature, human dental plaque is responsible for a variety of oral diseases and infections (Wood et al., 2000). Eradication or modification of dysbiotic biofilms is one of the primary treatment goals.

The use of antimicrobial solutions plays a key role in biofilm eradication among the antiplaque strategies by detaching and dissolving the biofilms and by facilitating the killing of biofilm microorganisms (Haapasalo et al., 2010; Howlin et al., 2015; Lecic et al., 2016). Because much of the biofilm consists of extracellular polymeric substance (EPS) (Xiao et al., 2012), an effective EPS dissolving process may facilitate biofilm eradication. Although different disinfecting solutions have been used in dental clinics, currently there are limited minimally invasive methods to evaluate and quantitatively measure their ability to dissolve biofilm bacteria and EPS (Sule et al., 2009; Corte et al., 2019).

Sodium hypochlorite (NaOCl) is the most commonly used irrigating solution in root canal treatment (Haapasalo et al., 2014). Chlorhexidine digluconate (CHX) has been recognized as the primary agent for chemical plaque control on tooth surfaces. Previous studies have set up different biofilm models to measure the magnitude of killing of biofilm bacteria by NaOCl and CHX (Ma et al., 2011; Stojicic et al., 2013). However, there is little data so far about the ability of NaOCl to dissolve biofilm or the dynamics of biofilm reaction to NaOCl or CHX (Diogo et al., 2017). One of the major challenges has been to establish an accurate *in vitro* assessment of their effects on biofilms (Montelongo-Jauregui et al., 2016). A recent study used a time-lapse killing assay to evaluate the effect of essential oil-based antimicrobial agents on single-species and three-species oral biofilms (Ren et al., 2019). Nevertheless, oral infections are caused by a mixed biofilm community acting as a multicellular organism embedded in EPS (Marsh and Zaura, 2017). There are no data available about the dynamic effectiveness of antibiofilm strategies against multispecies dental plaque biofilm.

The application of a novel protocol that allows real-time analysis of biofilm volume changes with fluorescent staining could be useful in obtaining, for the first time, real-time data on the dissolution of oral multispecies biofilm and inhibition of biofilm growth by antimicrobial agents. The present study aims to establish a standardized model to non-invasively evaluate the effect of NaOCl and CHX on oral multispecies biofilm.

## Materials and Methods

### Biofilm Culturing

Sterile hydroxyapatite (HA) disks (9.65 mm diameter × 1.52 mm thickness; Clarkson Chromatography Products, Williamsport, PA, United States) were used as the dental plaque biofilm culturing substrate. Biofilms were grown on the HA disks using a previously established model (Shen et al., 2010). The HA disks were coated with bovine dermal type I collagen (10 mg/mL collagen in 0.012M HCl in water) (Cohesion, Palo Alto, CA, United States) by overnight incubation at 4°C in 24-well tissue culture plates (Corning, Inc., Corning, NY, United States).

This study was approved by the Clinical Research Ethics Committee review boards in the university (certificate H12-02430). Subgingival plaque was collected from a systemically healthy adult volunteer in the age group of 20–50 years at 9 a.m. in the morning using sterilized wooden toothpicks. The plaque was suspended in brain–heart infusion broth (BHI; Becton Dickinson, Sparks, MD, United States) by pipetting. The bacterial suspension was adjusted to optical density (OD) = 0.10, which was measured in a 96-well microtiter plate (150 μL, 405 nm) by a microplate reader (Model 3350; Bio-Rad Laboratories, Richmond, CA, United States). The collagen-coated HA disks were placed in the 24-well tissue culture plates, each well-containing 1.8 mL of sterile BHI and 0.2 mL of dispersed plaque suspension. The disks were incubated in the BHI-plaque suspension under anaerobic conditions (AnaeroGen, OXOID, Hampshire, United Kingdom) at 37°C for 3 days or 3 weeks. The medium was changed once a week for the 3-week-old biofilm samples according to a previously described protocol (Shen et al., 2011).

### Dynamic Analysis of Biofilm Dissolution

After 3 days or 3 weeks of anaerobic incubation in BHI broth, a total of 32 biofilm samples from each of the 3-day-old and 3-week-old biofilm groups were used for the dynamic analysis of biofilm dissolution. The biofilm specimens were gently rinsed in 0.85% physiological saline for 15 s to remove the culture broth. The biofilm specimens were stained by a LIVE/DEAD BacLight Bacterial Viability kit L-7012 (Molecular Probes, Eugene, OR, United States), containing two component dyes (SYTO 9 and propidium iodide in a 1:1 mixture) following the manufacturer’s instructions. The bottom of the HA disks was then dried on paper (Kimwipes, Irving, TX, United States). The stained young (3-day-old) and old (3-week-old) biofilms were then exposed to the sterile water control, 2 or 6% NaOCl (EMD Chemicals, Inc., Darmstadt, Germany), or 2% CHX (Sigma Chemical, Corp., St. Louis, MO, United States) by gently adding 100 μL of medicaments on top of the biofilm. Each group contained eight samples. Then the biofilm sample was transferred to a glass-bottom Petri dish (35-mm diameter Petri dish with 14-mm microwell; MatTek, Corp., Ashland, MA, United States) for the live-cell imaging confocal laser scanning microscopy (LC-CLSM) (FV10i-LIV, Olympus, ON, Canada) analysis.

The excitation/emission maxima for the dyes were 480/500 nm for SYTO 9 and 490/635 nm for propidium iodide. Fluorescence from the stained cell was viewed using the LC-CLSM (FV10i-LIV, Olympus, ON, Canada) equipped with four laser diodes (405, 473, 559, 635 nm) at a resolution of 512 × 512 pixels and a scanning area of 500 μm × 500 μm using a 10 × lens. A random area of biofilm on each disk was scanned. A stack of slices in a 2-μm step-size Z axis was captured from the top to the bottom of the biofilm. The time between medicament placement and the start of confocal scanning was controlled within 2 min. The total length of live-cell imaging scanning was set for 30 min with repetitive scanning of the entire thickness of the biofilm with scanning cycles of 2 min.

Confocal images in the 30-min time lapse were then analyzed using Imaris 7.2 software (Bitplane, Inc., Saint Paul, MN, United States). The software reconstructed the two-dimensional intensity of fluorescence of all scanned layers into a three-dimensional volume stack at each cycle of scanning. The red (killed cells) and green (viable cells) fluorescence signals were separated by color threshold and the biovolume covered by each segmented color was calculated. The total biovolume of the biofilms was the sum of the volumes of green and red fluorescence.

The proportions of dissolved and killed biofilm were calculated as follows: dissolved biofilm % = (biofilm volume in sterile water group – biofilm volume in treatment group) × 100%/biofilm volume in sterile water group. Killed bacterial cells in residual biofilm % = (biofilm volume of red fluorescence/total biofilm volume of green and red fluorescence) × 100%. Dissolved and killed residual bacteria % = dissolved biofilm % + (1 – dissolved biofilm %) × killed bacterial cells in residual biofilm %.

### SEM Examination

The morphology of the biofilm bacteria after disinfection and dissolution were observed by scanning electron microscopy (SEM). Two additional 3-day-old or 3-week-old biofilm samples were subjected to a 100-μL droplet of sterile water, 2% NaOCl, 6% NaOCl, or 2% CHX using the same protocol as mentioned above for 10 min followed by rinsing in saline for 1 min. Samples were then prefixed with 2.5% glutaraldehyde for 10 min and further fixation in 1% osmium tetroxide for 1 h. The specimens were then subjected to increasing concentrations of ethanol (50, 70, 80, and 100%) for dehydration. The dehydrated specimens were dried using a critical point drier (Samdri-795; Tousimis Research Corporation, Rockville, MD, United States), sputter-coated with iridium (Leica EM MED020 Coating System, Tokyo, Japan), and examined by SEM (Helios Nanolab 650, FEI, Eindhoven, Netherlands) on two randomly selected areas on each sample using low (1,000×) and high (8,000×) magnifications at an accelerating voltage of 3 kV.

### EPS Dissolution

The EPS dissolution was examined by fluorescent labeling and LC-CLSM. A total of 16 biofilm samples from each of the 3-day-old and 3-week-old biofilm groups were prepared as described in Section “Biofilm Culturing” above for dynamic analysis of EPS dissolution. One μM Alexa Fluor 647-labeled dextran conjugate (Molecular Probes, Invitrogen, Corp., Carlsbad, CA, United States) was added to the BHI culture medium from the beginning of the development of the plaque biofilms according to a previously described protocol to stain the EPS (Xiao et al., 2012). The 3-day-old and 3-week-old biofilms were exposed to sterile water (control), 2 or 6% NaOCl, or 2% CHX by gently adding 100 μL of medicament solution on top of the biofilm. Each group contained four samples. Then each of the biofilm samples was transferred to a glass-bottom Petri dish for LC-CLSM (FV10i-LIV, Olympus, ON, Canada) analysis.

CLSM images of the biofilms were acquired by the LC-CLSM at a resolution of 512 × 512 pixels using a 10 × lens. The excitation/emission maxima for Alexa Fluor 647 was 647/668 nm. A random area of biofilm on each disk was scanned. A stack of slices in a 2-μm step-size Z axis was captured from the top to the bottom of the biofilm. The time between medicament placement and the start of confocal scanning was standardized to 2 min. The total length of live-cell imaging scanning was set for 30 min (total 32 min) with repetitive scanning of the entire thickness of the biofilm. The biovolume change of EPS was quantitated and analyzed by Imaris 7.2 software as mentioned above.

### Colony Forming Unit (CFU) Test

#### CFU for Dispersed Biofilm

The 3-day-old and 3-week-old biofilms on HA disk surfaces were scraped off into sterile water using a plastic loop and dispersed by pipetting and vortexing. The suspension was adjusted to an OD_405_ of 0.25 corresponding to 1.13 × 10^8^ CFU mL^–1^ as determined by serial 10-fold dilutions and anaerobic culturing on blood agar plates for colony forming unit (CFU) counts. A 100-μL sample of the dispersed plaque suspension was added to 400 μL of sterile water, 2% NaOCl, 6% NaOCl, or 2% CHX for 30 s or 2, 4, or 10 min. At each of the indicated times of exposure, 100 μL of bacterial solution was added to 900 μL BHI medium and diluted serially in 10-fold steps. The first two tubes in the dilution series contained inactivator (3% Tween 80 and 0.3% alpha-lecithin) (Sigma-Aldrich, St. Louis, MO, United States) for the CHX group and 0.5% sodium thiosulfate (Fisher Scientific, Ottawa, ON, Canada) for the NaOCl groups to reduce the carryover effect. Twenty μL from the dilution tubes was spotted onto blood agar plates (BHI agar with 5% heparinized sheep’s blood; Difco, Detroit, MI, United States). The blood agar plates were cultured anaerobically at 37°C for 48 h, and the CFU count was calculated. The number of CFU was generated as follows: (number of bacterial colonies in one 20-μL droplet) × 50 × 5 × 10 ^number of 10–fold dilutions–1^. The percentage of killed bacteria was defined as the difference between the percentage of living bacterial cells in the initial inoculum and that after exposure to the disinfecting solutions.

#### CFU for Intact Biofilm

The percentage of killed plaque biofilm bacteria after exposure to the agents without dispersion of the biofilm was also calculated in an additional experiment. The 3-day-old and 3-week-old biofilms on HA disks were exposed to sterile water, 2 or 6% NaOCl, or 2% CHX by dropping 100 μL of medicament solution on top of the biofilm for 10 min. The NaOCl-treated samples were rinsed in 1 mL of 0.5% sodium thiosulfate solution, and the CHX-treated samples were rinsed in 1 mL of CHX inactivator for 1 min. All the samples were rinsed in 1 mL sterile water for 1 min. Then the biofilms were scraped off into 1 mL sterile water and the OD_405_ value was adjusted to 0.25. A 100-μL sample of the plaque suspension was added to 400 μL of sterile water. A 100-μL sample of the bacterial solution was added to 900 μL BHI medium and diluted serially in 10-fold steps. Twenty μL from the dilution tubes was spotted onto blood agar plates. The blood agar plates were cultured anaerobically at 37°C for 48 h. The percentage of killed bacteria was calculated as stated above. All CFU experiments were performed in triplicate.

### Biofilm Inhibition

Sixteen new HA disks were coated with bovine dermal type I collagen as described in Section “Biofilm Culturing.”. Following collagen coating, the HA disks were immersed in a 2-mL solution of sterile water, 2% CHX, 0.2% CHX, or 0.02% CHX for 3 min with four samples each in 24-well plates. The HA disks were washed in sterile water for 1 min. Fresh dental plaque was collected using the same protocol mentioned in Section “Biofilm Culturing.” One hundred and fifty microliters of BHI-plaque suspension with the addition of 50 μL LIVE/DEAD BacLight viability stain was transferred onto the coverslip of the glass-bottom Petri dish (MatTek, Corp., Ashland, MA, United States). The CHX-coated HA disks were transferred onto the glass bottom (CHX-coated surface facing the plaque suspension) and the Petri dish was immediately subjected to LC-CLSM scanning at 37°C. A random area on the HA disk was selected with 512 × 512 pixels using a 10 × lens. A stack of 40 slices in 2-μm step sizes was captured in the area of interest. A total scanning time of 12 h with a full scan cycle of every 20 min was applied. Confocal images in the 12-h time lapse were quantitated and analyzed using Imaris 7.2 software (Bitplane, Inc., Saint Paul, MN, United States) as mentioned above. The total biovolume in the CHX and sterile water control groups at different time points was determined.

### Statistical Analysis

Statistical analysis was performed with the use of SPSS 16.0 (SPSS, Inc., Chicago, IL, United States) for Windows. Means and standard deviations of the biofilm biovolume and the proportions of dead cell volume were calculated. The normality of distribution was ensured by the Kolmogorov–Smirnov test and the homogeneity of variance was determined using Levene’s test.

Two-way repeated measures ANOVA was applied to determine the significance of the differences in biovolume and proportion of dissolved and killed bacteria, considering treatment as the main effect and treatment time as the repeated measure. Two-way ANOVA was applied to determine the significance of differences in the CFU counts. *Post hoc* multiple comparisons were used to isolate and compare the significant results using the Tukey test at a 5% significance level.

## Results

### Dynamics of Biofilm Dissolution and Microbial Killing

#### Three-Day-Old Biofilm

**FIGURE 1:**
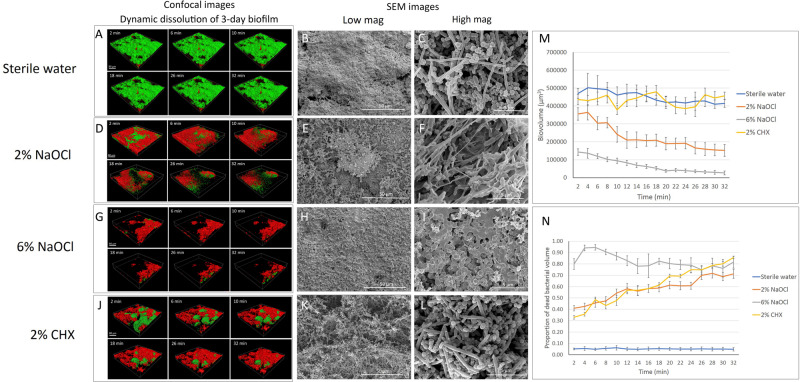
Three-day-old plaque biofilm treated by different disinfecting solutions (Green: live bacteria; red: killed bacteria; black: no bacteria). The six 3-D biofilm constructions from CLSM scannings were captured at 2, 6, 10, 18, 26, and 32 min of exposure to the indicated agents. **(A)** LC-CLSM images of killing of biofilm microbes and dynamic dissolution of biofilm during 32-min exposure to sterile water. SEM micrographs of biofilm exposure to sterile water under low **(B)** and high **(C)** magnifications. **(D)** LC-CLSM images of killing of biofilm microbes and dynamic dissolution of biofilm during 32-min exposure to 2% NaOCl. SEM micrographs of biofilm exposure to 2% NaOCl under low **(E)** and high **(F)** magnifications. **(G)** LC-CLSM images of killing of biofilm microbes and dynamic dissolution of biofilm during 32-min exposure to 6% NaOCl. SEM micrographs of biofilm exposure to 6% NaOCl under low **(H)** and high **(I)** magnifications. **(J)** LC-CLSM images of killing of biofilm microbes and dynamic dissolution of biofilm during 32-min exposure to 2% CHX. SEM micrographs of biofilm exposure to 2% CHX under low **(K)** and high **(L)** magnifications. **(M)** Biovolume of the biofilm during exposure to different endodontic disinfecting agents for 32 min. **(N)** The proportion of killed biofilm microbial cells in the residual biofilm during exposure to endodontic disinfecting agents for 32 min.

The time-dependent dynamic biofilm dissolution of 3-day-old biofilm is shown in Figure 1 and Supplementary Datasheet 2. No statistically significant differences (*P* > 0.05) in biofilm biovolume were observed in the sterile water group or the 2% CHX group during the 32-min exposure (Supplementary Table 1 and Figures 1A,J,M). Both 2 and 6% NaOCl (Figures 1D,G) significantly (*P* < 0.001) reduced the biovolume of the 3-day-old biofilm during the 32-min exposure (Supplementary Table 1 and Figure 1M). During the first 2 min, 6% NaOCl dissolved biofilm (70%) faster than 2% NaOCl (24%) (Supplementary Table 1). The proportion of dissolved biofilm increased significantly with time (*P* < 0.001) and only 6% of the biofilm remained in the 6% NaOCl group after 32 min.

With no antibacterial treatment, only 5 to 6% of the bacteria were stained with propidium iodide (PI), indicating dead bacteria (Supplementary Table 2). Forty-one percent of the bacteria in the undissolved biofilm were killed during the first 2 min of the 2% NaOCl treatment, and the percentage of killed bacteria gradually increased to 72% (*P* < 0.001) by 32 min (Supplementary Table 2 and Figures 1D,N). In comparison, 6% NaOCl resulted in a significantly higher proportion of killed biofilm bacteria (Supplementary Table 2 and Figures 1G,N). The percentage of killed bacteria in the non-dissolved biofilm in the 6% NaOCl group increased to a peak of 95% already by 6 min followed by a drop down to less than 80% after 32 min (*P* < 0.001) (Supplementary Table 2). In the CHX group, the amount of killed bacteria steadily increased from 33 to 86% during the 32-min treatment (Supplementary Table 2). No statistically significant difference was found in the killing of the bacteria between the 2% NaOCl and 2% CHX solutions (*P* > 0.05).

When the data for dissolved and killed bacteria in residual biofilm was combined, 6% NaOCl showed the highest antimicrobial effect with 94% bacterial reduction in 2 min and 99% in 32 min (*P* < 0.001) (Supplementary Table 3). Similarly, when the data for dissolved and killed bacteria were combined, 2% NaOCl resulted in a significantly higher bacterial reduction than 2% CHX in 32 min (*P* < 0.001) (Supplementary Table 3).

#### Three-Week-Old Biofilm

**FIGURE 2:**
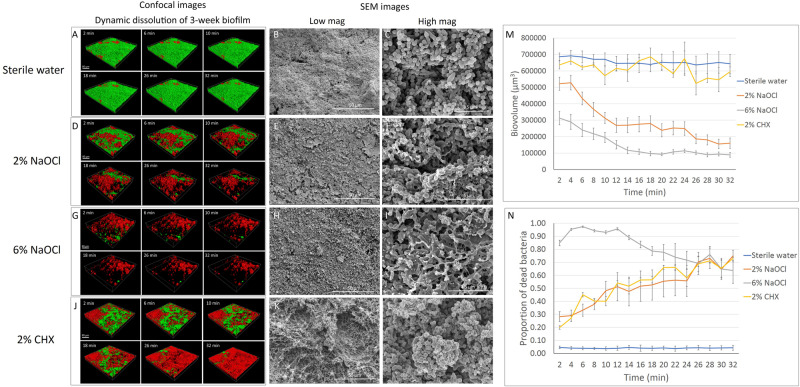
Three-week-old plaque biofilm treated by different disinfecting solutions (Green: live bacteria; red: killed bacteria; black: no bacteria). The six 3-D biofilm constructions from CLSM scannings were captured at 2, 6, 10, 18, 26, and 32 min of exposure to the indicated agents. **(A)** LC-CLSM images of killing of biofilm microbes and dynamic dissolution of biofilm during 32-min exposure to sterile water. SEM micrographs of biofilm exposure to sterile water under low **(B)** and high **(C)** magnifications. **(D)** LC-CLSM images of killing of biofilm microbes and dynamic dissolution of biofilm during 32-min exposure to 2% NaOCl. SEM micrographs of biofilm exposure to 2% NaOCl under low **(E)** and high **(F)** magnifications. **(G)** LC-CLSM images of killing of biofilm microbes and dynamic dissolution of biofilm during 32-min exposure to 6% NaOCl. SEM micrographs of biofilm exposure to 6% NaOCl under low **(H)** and high **(I)** magnifications. **(J)** LC-CLSM images of killing of biofilm microbes and dynamic dissolution of biofilm during 32-min exposure to 2% CHX. SEM micrographs of biofilm exposure to 2% CHX under low **(K)** and high **(L)** magnifications. **(M)** Biovolume of the biofilm during exposure to different endodontic disinfecting agents for 32 min. **(N)** The proportion of killed biofilm microbial cells in the residual biofilm during exposure to endodontic disinfecting agents for 32 min.

No significant change in biovolume was observed in the 3-week-old biofilm during the 32-min exposure to sterile water (Figures 2A,M). Similar to the effect on the 3-day-old biofilm, 2 and 6% NaOCl significantly dissolved the 3-week-old biofilm (Figures 2D,G,M) whereas 2% CHX had no dissolving effect (Figures 2J,M and Supplementary Datasheet 2). During the first 10 min of treatment, a significantly lower proportion of 3-week-old biofilm volume was dissolved by 6% NaOCl than previously measured for the 3-day-old biofilm (Supplementary Tables 1, 4) (*P* < 0.05). No significant difference was found in the reduction of biovolume by the 2% NaOCl solution for the 3-day-old and 3-week-old biofilms (*P* > 0.05).

Only 4–5% of the bacteria were stained with PI in the 3-week-old biofilm exposed to sterile water (Supplementary Table 5). Two percent NaOCl killed 28% of the residual 3-week-old biofilm in 2 min and 38% in 8 min (Supplementary Table 5). These results were both significantly lower than those obtained for the dissolution of the 3-day-old biofilm (Supplementary Table 2). Six percent NaOCl resulted in a similar pattern of killing for the 3-week-old and 3-day-old biofilms, reaching the highest killing effect of 97% at 6 min (Figures 2G,N and Supplementary Tables 2, 5). Two percent CHX showed a continuously increasing killing during the 32 min for the 3-week-old biofilm. The percentage of bacteria killed by CHX was 73% in 3-week-old biofilm, lower than in 3-day-old biofilm (86%) (*P* < 0.001) (Supplementary Tables 2, 5).

The total dissolved biofilm and killed residual bacteria in the 3-week-old biofilm showed no significant difference (*P* > 0.05) compared to the 3-day-old biofilm for 2 and 6% NaOCl (Supplementary Tables 3, 6). Two percent NaOCl resulted in almost the same level of bacterial reduction (94%) as that found in the 6% NaOCl group (95%) after 32 min (Supplementary Table 6). For 2% CHX, a significantly smaller proportion of dissolved biofilm and killed residual bacteria was found in the 3-week-old biofilm samples than in the 3-day-old biofilm samples (*P* < 0.01).

### Effect on Biofilm Structure

#### Three-Day-Old Biofilm

Biofilm exposed to water only showed a well-organized network structure with cocci, rods, and filaments within the biofilms (Figures 1B,C, 2B,C). Two percent NaOCl partially dissolved the 3-day-old biofilms and exposed the HA disk surface under the biofilm (Figure 1E). Disrupted biofilm bacterial cells and matrix could be observed under high magnification (Figure 1F). Almost no original biofilm structure could be detected after treatment by 6% NaOCl for 10 min (Figure 1H). High magnification SEM showed dispersed bacterial cells on the HA disk surfaces (Figure 1I). While an intact multispecies biofilm layer was shown after a 10-min treatment by 2% CHX (Figure 1K), small particles were observed on the bacterial cell surfaces as a result of partial cell lysis (Figure 1L).

#### Three-Week-Old Biofilm

The dissolution of biofilm by 2% NaOCl resulted in empty areas (gaps) within the 3-week-old biofilm layer (Figure 2E) and bacterial cell rupture (Figure 2F). Six percent NaOCl dissolved most of the 3-week-old biofilm and exposed HA disk surface (Figures 2H,I). Two percent CHX resulted in a similar cell lysis effect for both the 3-day-old and 3-week-old biofilms (Figures 2K,L).

### EPS Dissolution

**FIGURE 3:**
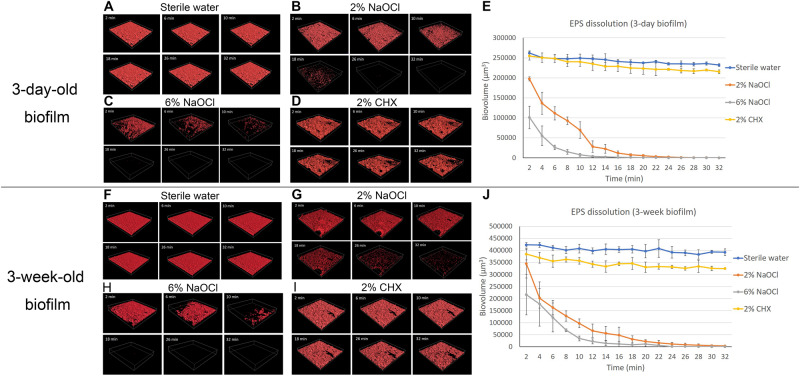
LC-CLSM images of EPS biovolume of 3-day-old and 3-week-old biofilms during 32-min exposure to different disinfecting solutions: **(A)** sterile water on 3-day-old plaque. **(B)** 2% NaOCl on 3-day-old plaque. **(C)** 6% NaOCl on 3-day-old plaque. **(D)** 2% CHX on 3-day-old plaque. **(E)** Biovolume of the EPS of the 3-day-old biofilm during exposure to different endodontic disinfecting agents for 32 min. **(F)** Sterile water on 3-week-old plaque. **(G)** 2% NaOCl on 3-week-old plaque. **(H)** 6% NaOCl on 3-week-old plaque. **(I)** 2% CHX on 3-week-old plaque. **(J)** Biovolume of the EPS of the 3-week-old biofilm during exposure to different endodontic disinfecting agents for 32 min.

Sterile water did not change the biovolume of EPS during the 32-min exposure (Figures 3A,E,F,J). Two percent NaOCl (Figures 3B,G) and 6% NaOCl (Figures 3C,H) significantly reduced the biovolume of EPS in the 3-day-old and 3-week-old biofilms after 32 min (*P* < 0.001) (Supplementary Datasheet 2). A rapid decrease in biovolume was observed from 2 to 10 min for both NaOCl groups (*P* < 0.001). Two and six percent NaOCl reduced the EPS biovolume by over 70 and 90%, respectively, in 10 min for both the 3-day-old and 3-week-old biofilms. Sodium hypochlorite gradually dissolved almost all EPS (>99.9%) in 32 min. Two percent CHX reduced the biovolume of EPS of the 3-day-old and 3-week-old plaque biofilm only slightly in 32 min (Figures 3D,E,I,J) (*P* < 0.05).

### Bacterial Killing Measured by CFU

All three solutions killed the plaque biofilm bacteria within 10 min in the planktonic state (biofilm was dispersed) (Table 1). Over 99% of the bacteria were killed in 30 s by 6% NaOCl (Table 1). Six percent NaOCl killed microbes faster than 2% NaOCl and 2% CHX by killing all bacteria from 3-day-old dispersed biofilm in 2 min (*P* < 0.01) and from 3-week-old dispersed biofilm in 4 min (*P* < 0.01). For the 3-week-old biofilms, 2% NaOCl and 2% CHX killed 99.82 and 99.50%, respectively, of the dispersed bacteria in 4 min (Table 1).

**TABLE 1:** Percentage (% ± standard deviation) of killed plaque biofilm bacteria in dispersed biofilm and in intact biofilm after exposure to the three medicaments.

		**Dispersed biofilm**				**Intact biofilm**
Time (min)	Biofilm age	30 s^e^	2 min^f^	4 min^g^	10 min^h^	10 min^i^
2% NaOCl	3-day^a^	94.62 ± 1.84	99.77 ± 0.13	100.00 ± 0.00	100.00 ± 0.00	97.44 ± 1.13
	3-week^a^	95.73 ± 1.57	98.81 ± 0.42	99.82 ± 0.06	100.00 ± 0.00	94.33 ± 1.48
6% NaOCl	3-day^b^	99.71 ± 0.11	100.00 ± 0.00	100.00 ± 0.00	100.00 ± 0.00	100.00 ± 0.00
	3-week^b^	99.54 ± 0.18	99.96 ± 0.02	100.00 ± 0.00	100.00 ± 0.00	99.76 ± 0.12
2% CHX	3-day^c^	92.57 ± 2.18	99.25 ± 0.18	100.00 ± 0.00	100.00 ± 0.00	98.10 ± 1.07
	3-week^d^	90.99 ± 1.84	94.37 ± 2.42	99.50 ± 0.11	100.00 ± 0.00	96.82 ± 1.74

*Different superscript letters indicate statistical differences between groups (*P* < 0.05). Two-way ANOVA was applied for statistical analysis.*

Only 6% NaOCl was able to kill all bacteria in the 3-day-old undispersed biofilm (Table 1). Two percent NaOCl and 2% CHX achieved a 97–99% killing in 3-day-old biofilm and a 94–97% killing in 3-week-old biofilm. Six percent NaOCl showed the strongest antibiofilm activity, although the 3-week-old biofilm was more resistant to all three solutions than the 3-day-old biofilm (*P* < 0.01) (Table 1).

### Inhibition of Biofilm Growth

**FIGURE 4:**
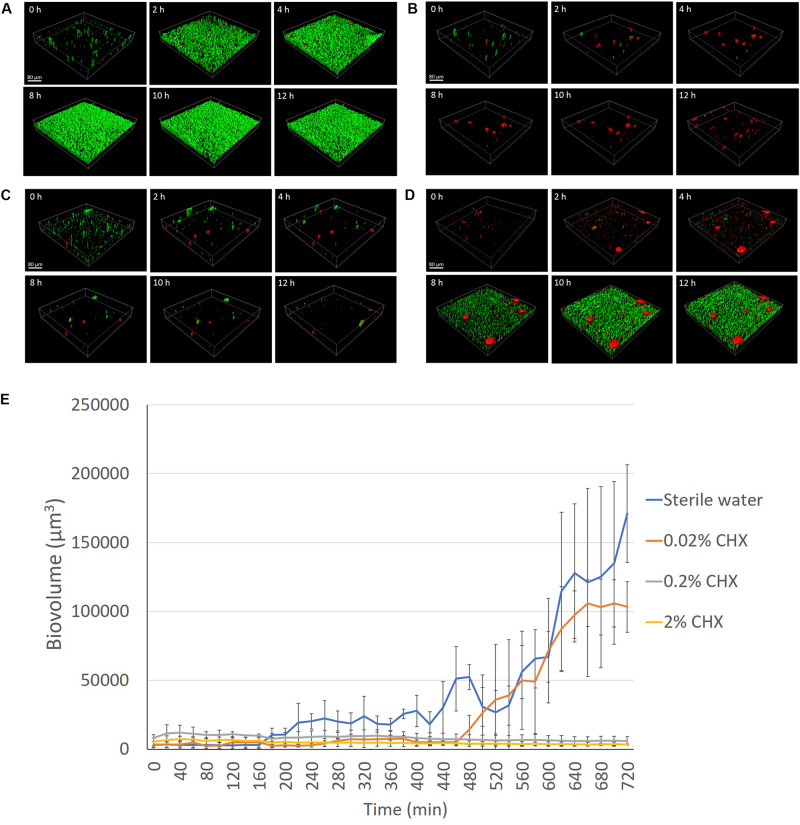
LC-CLSM images of biofilm development during the first 12 h of biofilm incubation at indicated times on HA disks pretreated for 3 min with sterile water **(A)**, 2% CHX **(B)**, 0.2% CHX **(C)**, and 0.02% CHX **(D)**. **(E)** Plaque biofilm growth on different concentrations (2, 0.2, and 0.02%) of CHX-coated HA disk for 12 h (720 min).

The biovolume of dental plaque bacteria grown on the HA disks in BHI broth remained extremely low and stable when pretreated with 2 or 0.2% CHX during the 12-h (720-min) culturing period (*P* > 0.05) (Figures 4B,C,E and Supplementary Datasheet 2). The biovolume of the plaque biofilm started to increase significantly (*P* < 0.001) by 4 h in the control disks not pretreated with CHX (Figures 4A,E). Plaque on the sterile water-coated HA disks reached a biovolume level that was 53 times higher after 12 h than the biovolume of fresh plaque detected on the HA surfaces at the beginning of the biofilm culture (0 h) (Figure 4E). The plaque biovolume on the 0.02% CHX-coated HA disk did not increase in the first 8 h (480 min) (*P* > 0.05) (Figure 4D). However, a rapid increase in biovolume (*P* < 0.001) was observed on the disks from 8 to 11 h (660 min) (Figures 4D,E).

## Discussion

Dental plaque is a dynamic microbial biofilm ecosystem that comprises 100s of species. A recent study used pyrosequencing to assess 160 supragingival plaque samples and showed that the dominant phyla of plaque microbiota were *Bacteroidetes*, *Actinobacteria*, *Firmicutes*, *Proteobacteria*, and *Fusobacteria*. The dominant genera included *Capnocytophaga*, *Prevotella*, *Actinomyces*, *Streptococcus*, *Neisseria*, *Corynebacterium*, *Rothia*, and *Leptotrichia* (Xiao et al., 2016). The succession of the dental plaque development takes place in an ordered sequence of events with facultative and aerobic bacteria predominantly colonizing the tooth surface in the early stages, and the proportions of facultative and anaerobic genera increasing with plaque maturation (Takeshita et al., 2015).

Sodium hypochlorite and chlorhexidine were chosen as the antimicrobial agents in the present study because they are the most commonly used antibacterial agents in dentistry (Haapasalo et al., 2005; Gunsolley, 2010). Multispecies dental plaque biofilm models have been used in previous studies to test the effect of antimicrobial agents (Shen et al., 2009, 2010). Another study used CLSM and acridine orange stain to analyze biofilm dissolution on bovine dentin by CHX and NaOCl (Del Carpio-Perochena et al., 2011). However, a two-dimensional analysis was performed, and fixed treatment time intervals were applied in this model. A more recent study showed time-lapse killing assay performed using real-time bacterial staining and imaging using single-species (*Streptococcus mutans*) and three-species (*S. mutans, Actinomyces naeslundii*, and *Streptococcus oralis*) biofilms. It has been reported that around 30–40% of the dry weight of dental plaque is composed of polysaccharides (Costa Oliveira et al., 2017). Nevertheless, little information has been provided on the dynamic dissolution of EPS in multi-species plaque biofilms. Microbial cells with intact cell membranes stain fluorescent green, whereas those with damaged membranes stain fluorescent red using the LIVE/DEAD viability stain (Zhang et al., 2015). Sterile water control groups in the present study showed that there was no significant change in biofilm biovolume during the 32-min exposure, and the percentage of dead bacteria (as indicated by red fluorescence from the PI) was 4–6% (Figures 1, 2), indicating that neither the viability stain nor any other environmental factor in the setting induced biofilm dissolution or killing. Moreover, in the biofilm inhibition test, the sterile water control group showed normal biofilm growth (Figure 4), indicating that the viability stain and sterile water did not inhibit biofilm growth on the collagen-coated HA disks.

The dynamic live-cell imaging CLSM showed strong biofilm dissolution, EPS dissolution, and bacterial killing by NaOCl. The EPS of the plaque biofilm is mostly composed of proteins and extracellular DNA (Xiao et al., 2012). The mechanism of action of NaOCl is mainly due to its oxidizing effect and high pH (Fukuzaki, 2006). Hypochlorous acid (HOCl) and hypochlorite ions (OCl^–^) from NaOCl lead to amino acid degradation and hydrolysis. The dissolving capability of NaOCl relies on its concentration and contact time. A previous study analyzing two-dimensional CLSM images showed that 5.25% NaOCl dissolved a significantly higher amount (97%) of 3-day-old oral plaque biofilm than 2.5% NaOCl (39.5%) in 5 min, but there was no difference between them after 30 min (Del Carpio-Perochena et al., 2011). This result is partially consistent with the present study by showing that a higher concentration of NaOCl had a higher biofilm dissolving ability. The current study found that 2% NaOCl dissolved a significantly lower amount of biofilm than 6% NaOCl in 32 min, which may be due to less physicochemical interactions between the OCl^–^ and EPS/bacterial cells in the 2% NaOCl group. The difference between the two studies could be due to the different experimental design. It is interesting to observe that biofilms in the present study were not 100% dissolved or killed in the 32-min exposure to disinfecting agents (Supplementary Tables 3, 6). However, the EPS was almost 100% dissolved by 2 and 6% NaOCl in 32 min (Figure 3). The reason for this is most likely that the limited amount of disinfecting solution (100 μL) could not completely dissolve the bacteria in the biofilm (Xiao et al., 2012; Liu et al., 2018). In the clinical situation of an infected root canal, it is likely that in different anatomical locations such as fins and anastomoses between canals, and at the apical foramen, the volume of irrigant in contact with the biofilm is very low (<2 μL) and exchange minimal (Huang et al., 2008; Gao et al., 2009). Pilot experiments indicated that adding more disinfecting solution can result in shifting of the biofilm sample on the Petri dish and making the confocal scanning unstable. Another possible explanation for the biofilm resistance is the existence of persister cells, which represent dormant or slow-growing bacteria in a population that resist the action of antimicrobial agents (Spoering and Lewis, 2001; Shen et al., 2016).

When the overall antibiofilm effect was calculated as biofilm dissolution added to the proportion of killed microbes in the residual biofilm, 2% NaOCl showed stronger effects than 2% CHX (Supplementary Tables 3, 6), whereas no difference was found between them when only the killed cell proportion in the residual biofilm was compared (Supplementary Tables 2, 5). This is due to the fact that NaOCl has both killing and dissolving effects whereas CHX only kills bacteria (Hope and Wilson, 2004; Del Carpio-Perochena et al., 2011). Although the SEM images showed different surface structural details for CHX and sterile water-treated biofilm, the biovolume of the biofilm was not different. The mechanism for the changes in biofilm surface topography in CHX-exposed biofilms may be due to ionic interactions between the negatively charged EPS matrix and the positive CHX, causing immediate collapse of the superficial matrix polysaccharides, resulting in a minor biovolume reduction (Jones, 1997; Shen et al., 2016) (Figure 3). The EPS matrix tends to hinder the diffusion of CHX into the deeper layers of biofilms (Shen et al., 2011). Up to 86 and 73% of the 3-day-old and 3-week-old biofilm bacteria, respectively, were killed in 32 min and biofilms treated with both 2% CHX and 2% NaOCl showed equal killing of residual biofilm microbes (Supplementary Tables 2, 5).

Clinically, in endodontics the use of CHX is considered to be advantageous prior to obturation due to its substantivity on the dentin surface (Rosenthal et al., 2004). Chlorhexidine on the dentin surface can remain antimicrobially active for up to 12 weeks (Rosenthal et al., 2004). The present study used three different concentrations of CHX to coat the HA disks and then monitored plaque biofilm growth under LC-CLSM for 12 h. Pre-exposure of the HA disks to 2 and 0.2% CHX inhibited biofilm growth for the entire 12 h, while the 0.02% CHX only delayed the start of biofilm growth (Figure 4). This result probably indicated that CHX achieved plaque inhibition as a result of an immediate bactericidal action followed by a prolonged bacteriostatic action due to adsorption to the HA disk surface (Jenkins et al., 1988). The effectiveness of this inhibitory activity is influenced by the concentration of CHX (Rosenthal et al., 2004). Lower concentration (0.02%) has lower amount of CHX to bind to the hydroxyapatite surface, which allows more surface attachment of the planktonic bacteria (Rosenthal et al., 2004). Another mechanism of the sub-inhibitory effect of low concentration of CHX could be due to the fact that the limited number of positively charged CHX molecules were not able to break down enough glycan chains of the matrix polysaccharides during the biofilm maturation (Shen et al., 2016), and thus the plaque biofilm was able to form in 12 h.

The present study is the first of its kind to observe and compare the dynamic dissolution and killing of young and old multispecies biofilms by antimicrobial solutions. A recent study showed that the proportion bacteria killed by CHX in mature biofilms was much lower than in young biofilms (Shen et al., 2011). This was consistent with the results obtained in the present study (Supplementary Tables 2, 4). Six percent NaOCl dissolved significantly more 3-day-old biofilm than 3-week-old biofilm, whereas 2% NaOCl removed a similar (but lower than 6% NaOCl) percentage of young and old biofilms (Supplementary Tables 1, 3), which can be explained by a stronger oxidizing effect from 6% NaOCl than the effect from 2% NaOCl.

As the type of fluorescence (green versus red) of the bacteria is based on cell membrane integrity rather than confirmed cell death, the killing as suggested by CLSM was further confirmed using culturing and CFU counting. The undispersed biofilm (intact biofilm) bacteria were more resistant than dispersed biofilm bacteria (Table 1), probably due to differences in their state of metabolic activity. The mechanisms for the resistance of biofilm include physical diffusion barriers, altered expression of resistance genes by the bacteria, and the emergence of biofilm-specific phenotypes (Davies, 2003; Shen et al., 2016).

Despite the advantages of LC- CLSM in the analysis of dynamic dissolution and inhibition of the oral biofilm, there are also limitations associated with the method utilized in the present study. Although all the biofilms in the present study were cultivated *in vitro*, future studies using biofilms cultivated *in vivo* should be considered to better mimic the clinical reality. There was a 2-min gap between applying the disinfecting solutions to the biofilm samples and the start of confocal scanning; thus the baseline (0 min) biovolume could not be captured. Therefore, this model may not be optimal for the assessment of agents that exhibit extremely rapid killing or dissolution of the biofilm. However, agents with more rapid action than 6% NaOCl may be difficult to find or use clinically. Using this model, various other disinfecting solutions used for biofilm modification, removal, or killing can be studied.

## Data Availability Statement

All datasets generated for this study are included in the article/Supplementary Material. Further inquiries about the raw data can be directed to the corresponding author.

## Ethics Statement

The studies involving human participants were reviewed and approved by The University of British Columbia. The patients/participants provided their written informed consent to participate in this study.

## Author Contributions

ZW contributed to conception, design, data acquisition and interpretation, performed all statistical analyses, and drafted and revised the manuscript. YS contributed to conception and revised the manuscript. MH contributed to conception, design, and critically revised the manuscript.

## Conflict of Interest

The authors declare that the research was conducted in the absence of any commercial or financial relationships that could be construed as a potential conflict of interest.

## References

[B1] Corte L. Casagrande Pierantoni D. Tascini C. Roscini L. Cardinali G. (2019). Biofilm specific activity: a measure to quantify microbial biofilm. *Microorganisms* 7 1–14. 10.3390/microorganisms7030073 PMC6463164 30866438

[B2] Costa Oliveira B. E. Cury J. A. Ricomini Filho A. P. (2017). Biofilm extracellular polysaccharides degradation during starvation and enamel demineralization. *PLoS ONE* 12:e0181168 10.1371/journal.pone.0181168 PMC5513492 28715508

[B3] Davies D. (2003). Understanding biofilm resistance to antibacterial agents. *Nat. Rev. Drug. Discov.* 2 114–122. 10.1038/nrd1008 12563302

[B4] Del Carpio-Perochena A. E. Bramante C. M. Duarte M. A. Cavenago B. C. Villas-Boas M. H. Graeff M. S. (2011). Biofilm dissolution and cleaning ability of different irrigant solutions on intraorally infected dentin. *J. Endod.* 37 1134–1138. 10.1016/j.joen.2011.04.013 21763908

[B5] Diogo P. Fernandes C. Caramelo F. Mota M. Miranda I. M. Faustino M. A. F. (2017). Antimicrobial photodynamic therapy against endodontic *Enterococcus faecalis* and *Candida albicans* mono and mixed biofilms in the presence of photosensitizers: a comparative study with classical endodontic irrigants. *Front. Microbiol.* 8:498 10.3389/fmicb.2017.00498 PMC5371592 28424663

[B6] Fukuzaki S. (2006). Mechanisms of actions of sodium hypochlorite in cleaning and disinfection processes. *Biocontrol. Sci.* 11 147–157. 10.4265/bio.11.147 17190269

[B7] Gao Y. Haapasalo M. Shen Y. Wu H. Li B. Ruse N. D. (2009). Development and validation of a three-dimensional computational fluid dynamics model of root canal irrigation. *J. Endod.* 35 1282–1287. 10.1016/j.joen.2009.06.018 19720232

[B8] Gunsolley J. C. (2010). Clinical efficacy of antimicrobial mouthrinses. *J. Dent.* 38(Suppl. 1), S6–S10. 10.1016/S0300-5712(10)70004-X 20621242

[B9] Haapasalo M. Endal U. Zandi H. Coil J. (2005). Eradication of endodontic infection by instrumentation and irrigation solutions. *Endod. Topics* 10 77–102. 10.1111/j.1601-1546.2005.00135.x

[B10] Haapasalo M. Shen Y. Qian W. Gao Y. (2010). Irrigation in endodontics. *Dent. Clin. N. Am.* 54 291–312. 10.1016/j.cden.2009.12.001 20433979

[B11] Haapasalo M. Shen Y. Wang Z. Gao Y. (2014). Irrigation in endodontics. *Br. Dent. J.* 216 299–303. 10.1038/sj.bdj.2014.204 24651335

[B12] Hope C. K. Wilson M. (2004). Analysis of the effects of chlorhexidine on oral biofilm vitality and structure based on viability profiling and an indicator of membrane integrity. *Antimicrob. Agents Chemother.* 48 1461–1468. 10.1128/aac.48.5.1461-1468.2004 15105093 PMC400577

[B13] Howlin R. P. Fabbri S. Offin D. G. Symonds N. Kiang K. S. Knee R. J. (2015). Removal of dental biofilms with an ultrasonically activated water stream. *J. Dent. Res.* 94 1303–1309. 10.1177/0022034515589284 26056055

[B14] Huang T. Y. Gulabivala K. Ng Y. L. (2008). A bio-molecular film *ex-vivo* model to evaluate the influence of canal dimensions and irrigation variables on the efficacy of irrigation. *Int. Endod. J.* 41 60–71. 10.1111/j.1365-2591.2007.01317.x 17916068

[B15] Jenkins S. Addy M. Wade W. (1988). The mechanism of action of chlorhexidine. *A study of plaque growth on enamel inserts in vivo*. *J. Clin. Periodontol.* 15 415–424. 10.1111/j.1600-051x.1988.tb01595.x 3183067

[B16] Jones C. G. (1997). Chlorhexidine: is it still the gold standard? *Periodontology* 2000 55–62. 10.1111/j.1600-0757.1997.tb00105.x 9643233

[B17] Lecic J. Cakic S. Janjic Pavlovic O. Cicmil A. Vukotic O. Petrovic V. (2016). Different methods for subgingival application of chlorhexidine in the treatment of patients with chronic periodontitis. *Acta. Odontol. Scand.* 74 502–507. 10.1080/00016357.2016.1206964 27409799

[B18] Leung K. P. Crowe T. D. Abercrombie J. J. Molina C. M. Bradshaw C. J. Jensen C. L. (2005). Control of oral biofilm formation by an antimicrobial decapeptide. *J. Dent. Res.* 84 1172–1177. 10.1177/154405910508401215 16304449

[B19] Liu Y. Ren Z. Hwang G. Koo H. (2018). Therapeutic strategies targeting cariogenic biofilm microenvironment. *Adv. Dent. Res.* 29 86–92. 10.1177/0022034517736497 29355421 PMC5784482

[B20] Ma J. Wang Z. Shen Y. Haapasalo M. (2011). A new noninvasive model to study the effectiveness of dentin disinfection by using confocal laser scanning microscopy. *J. Endod.* 37 1380–1385. 10.1016/j.joen.2011.06.018 21924186

[B21] Marsh P. D. Zaura E. (2017). Dental biofilm: ecological interactions in health and disease. *J. Clin. Periodontol.* 44(Suppl. 18), S12–S22. 10.1111/jcpe.12679 28266111

[B22] Montelongo-Jauregui D. Srinivasan A. Ramasubramanian A. K. Lopez-Ribot J. L. (2016). An *in vitro* model for oral mixed biofilms of *candida albicans* and *streptococcus gordonii* in synthetic saliva. *Front. Microbiol.* 7:686 10.3389/fmicb.2016.00686 PMC4864667 27242712

[B23] Ren Z. Kim D. Paula A. J. Hwang G. Liu Y. Li J. (2019). Dual-targeting approach degrades biofilm matrix and enhances bacterial killing. *J. Dent. Res.* 98 322–330. 10.1177/0022034518818480 30678538

[B24] Rosenthal S. Spangberg L. Safavi K. (2004). Chlorhexidine substantivity in root canal dentin. *Oral. Surg. Oral. Med. Oral. Pathol. Oral. Radiol. Endod.* 98 488–492. 10.1016/S1079210403004815 15472666

[B25] Shen Y. Qian W. Chung C. Olsen I. Haapasalo M. (2009). Evaluation of the effect of two chlorhexidine preparations on biofilm bacteria in vitro: a three-dimensional quantitative analysis. *J. Endod.* 35 981–985. 10.1016/j.joen.2009.04.030 19567319

[B26] Shen Y. Stojicic S. Haapasalo M. (2011). Antimicrobial efficacy of chlorhexidine against bacteria in biofilms at different stages of development. *J. Endod.* 37 657–661. 10.1016/j.joen.2011.02.007 21496666

[B27] Shen Y. Stojicic S. Qian W. Olsen I. Haapasalo M. (2010). The synergistic antimicrobial effect by mechanical agitation and two chlorhexidine preparations on biofilm bacteria. *J. Endod.* 36 100–104. 10.1016/j.joen.2009.09.018 20003944

[B28] Shen Y. Zhao J. de la Fuente-Nunez C. Wang Z. Hancock R. E. Roberts C. R. (2016). Experimental and theoretical investigation of multispecies oral biofilm resistance to chlorhexidine treatment. *Sci. Rep.* 6:27537 10.1038/srep27537 PMC4914838 27325010

[B29] Spoering A. L. Lewis K. (2001). Biofilms and planktonic cells of *Pseudomonas aeruginosa* have similar resistance to killing by antimicrobials. *J. Bacteriol.* 183 6746–6751. 10.1128/JB.183.23.6746-6751.2001 11698361 PMC95513

[B30] Stojicic S. Shen Y. Haapasalo M. (2013). Effect of the source of biofilm bacteria, level of biofilm maturation, and type of disinfecting agent on the susceptibility of biofilm bacteria to antibacterial agents. *J. Endod.* 39 473–477. 10.1016/j.joen.2012.11.024S0099-2399(12)01081-3 23522539

[B31] Sule P. Wadhawan T. Carr N. J. Horne S. M. Wolfe A. J. Pruss B. M. (2009). A combination of assays reveals biomass differences in biofilms formed by *Escherichia coli* mutants. *Lett. Appl. Microbiol.* 49 299–304. 10.1111/j.1472-765X.2009.02659.x 19552773 PMC2908736

[B32] Takeshita T. Yasui M. Shibata Y. Furuta M. Saeki Y. Eshima N. (2015). Dental plaque development on a hydroxyapatite disk in young adults observed by using a barcoded pyrosequencing approach. *Sci. Rep.* 5:8136 10.1038/srep08136 PMC4311255 25633431

[B33] Wood S. R. Kirkham J. Marsh P. D. Shore R. C. Nattress B. Robinson C. (2000). Architecture of intact natural human plaque biofilms studied by confocal laser scanning microscopy. *J. Dent. Res.* 79 21–27. 10.1177/00220345000790010201 10690656

[B34] Xiao C. Ran S. Huang Z. Liang J. (2016). Bacterial diversity and community structure of supragingival plaques in adults with dental health or caries revealed by 16s pyrosequencing. *Front. Microbiol.* 7:1145 10.3389/fmicb.2016.01145 PMC4956651 27499752

[B35] Xiao J. Klein M. I. Falsetta M. L. Lu B. Delahunty C. M. Yates J. R. III (2012). The exopolysaccharide matrix modulates the interaction between 3D architecture and virulence of a mixed-species oral biofilm. *PLoS Pathog.* 8:e1002623 10.1371/journal.ppat.1002623 PMC3320608 22496649

[B36] Zhang K. Wang S. Zhou X. Xu H. H. Weir M. D. Ge Y. (2015). Effect of antibacterial dental adhesive on multispecies biofilms formation. *J. Dent. Res.* 94 622–629. 10.1177/0022034515571416 25715378 PMC4485219

