# Supplementary material for: Dynamics of Dissolution, Killing, and Inhibition of Dental Plaque Biofilm
Source: Front Microbiol. 2020 May 20;11:964. doi: 10.3389/fmicb.2020.00964 (PMC7251032; doi:10.3389/fmicb.2020.00964)
Supplement: Supplementary file 1 [file Data_Sheet_1.docx]

### Supplementary Materials

**Supplementary Table** **1.** Proportion of Dissolved 3-day-old Plaque Biofilm during Exposure to Disinfecting Agents.

| Time (min) | 2% NaOCl^a^ | 6% NaOCl^b^ | 2% CHX^c^ |
| --- | --- | --- | --- |
| 2 | 0.24±0.05 | 0.70±0.05 | 0.07±0.10 |
| 4 | 0.26±0.11 | 0.73±0.05 | 0.13±0.14 |
| 6 | 0.38±0.08 | 0.76±0.04 | 0.09±0.15 |
| 8 | 0.37±0.06 | 0.79±0.04 | 0.05±0.14 |
| 10 | 0.48±0.08 | 0.79±0.04 | 0.17±0.07 |
| 12 | 0.55±0.11 | 0.82±0.03 | 0.08±0.13 |
| 14 | 0.55±0.10 | 0.85±0.03 | 0.06±0.15 |
| 16 | 0.54±0.07 | 0.86±0.02 | -0.03±0.15 |
| 18 | 0.52±0.08 | 0.88±0.02 | -0.11±0.13 |
| 20 | 0.55±0.09 | 0.91±0.02 | -0.02±0.13 |
| 22 | 0.55±0.08 | 0.90±0.02 | 0.06±0.14 |
| 24 | 0.54±0.08 | 0.91±0.02 | 0.07±0.12 |
| 26 | 0.61±0.09 | 0.92±0.02 | 0.07±0.10 |
| 28 | 0.63±0.08 | 0.93±0.02 | -0.09±0.12 |
| 30 | 0.62±0.09 | 0.93±0.03 | -0.09±0.12 |
| 32 | 0.63±0.08 | 0.94±0.03 | -0.10±0.09 |

Different superscript letters indicate statistical differences between groups (P < 0.05). Repeated measures ANOVA was applied for statistical analysis.

**Supplementary Table** **2.** Proportion of Killed Biofilm Microbial Cell Biovolume (PI-Stained Bacteria) in Residual 3-day-old Biofilm during Exposure to Disinfecting Agents.

| Time (min) | Sterile water^a^ | 2% NaOCl^b^ | 6% NaOCl^c^ | 2% CHX^b^ |
| --- | --- | --- | --- | --- |
| 2 | 0.05±0.01 | 0.41±0.02 | 0.80±0.05 | 0.33±0.02 |
| 4 | 0.05±0.02 | 0.42±0.03 | 0.94±0.03 | 0.36±0.02 |
| 6 | 0.05±0.01 | 0.46±0.04 | 0.95±0.02 | 0.49±0.02 |
| 8 | 0.06±0.02 | 0.47±0.04 | 0.91±0.02 | 0.43±0.03 |
| 10 | 0.06±0.02 | 0.54±0.04 | 0.87±0.03 | 0.48±0.04 |
| 12 | 0.05±0.01 | 0.58±0.05 | 0.83±0.04 | 0.57±0.02 |
| 14 | 0.05±0.01 | 0.56±0.04 | 0.78±0.07 | 0.57±0.04 |
| 16 | 0.05±0.02 | 0.58±0.03 | 0.78±0.10 | 0.58±0.03 |
| 18 | 0.05±0.01 | 0.59±0.03 | 0.82±0.03 | 0.61±0.02 |
| 20 | 0.05±0.01 | 0.61±0.03 | 0.80±0.05 | 0.69±0.02 |
| 22 | 0.05±0.01 | 0.61±0.04 | 0.79±0.04 | 0.69±0.02 |
| 24 | 0.05±0.02 | 0.61±0.03 | 0.79±0.07 | 0.75±0.03 |
| 26 | 0.05±0.02 | 0.70±0.04 | 0.75±0.04 | 0.75±0.03 |
| 28 | 0.05±0.02 | 0.72±0.04 | 0.78±0.07 | 0.79±0.02 |
| 30 | 0.05±0.01 | 0.69±0.03 | 0.76±0.08 | 0.80±0.02 |
| 32 | 0.05±0.01 | 0.71±0.04 | 0.82±0.05 | 0.86±0.01 |

Different superscript letters indicate statistical differences between groups (P < 0.05). Repeated measures ANOVA was applied for statistical analysis.

**Supplementary Table** **3.** Proportion of Dissolved and Killed Residual Bacteria (PI-Stained Bacteria) in 3-day-old Biofilm during Exposure to Disinfecting Agents.

| Time (min) | 2% NaOCl^a^ | 6% NaOCl^b^ | 2% CHX^c^ |
| --- | --- | --- | --- |
| 2 | 0.55±0.04 | 0.94±0.02 | 0.38±0.07 |
| 4 | 0.58±0.06 | 0.98±0.01 | 0.44±0.09 |
| 6 | 0.66±0.05 | 0.99±0.01 | 0.53±0.09 |
| 8 | 0.67±0.04 | 0.98±0.01 | 0.46±0.10 |
| 10 | 0.76±0.04 | 0.97±0.01 | 0.57±0.06 |
| 12 | 0.81±0.04 | 0.97±0.01 | 0.60±0.07 |
| 14 | 0.80±0.04 | 0.97±0.01 | 0.59±0.07 |
| 16 | 0.81±0.04 | 0.97±0.02 | 0.57±0.07 |
| 18 | 0.80±0.04 | 0.98±0.00 | 0.57±0.07 |
| 20 | 0.82±0.04 | 0.98±0.00 | 0.69±0.05 |
| 22 | 0.82±0.04 | 0.98±0.00 | 0.71±0.04 |
| 24 | 0.82±0.04 | 0.98±0.01 | 0.77±0.04 |
| 26 | 0.88±0.02 | 0.98±0.00 | 0.76±0.04 |
| 28 | 0.90±0.02 | 0.98±0.00 | 0.77±0.03 |
| 30 | 0.88±0.03 | 0.98±0.01 | 0.78±0.02 |
| 32 | 0.90±0.02 | 0.99±0.00 | 0.84±0.01 |

Different superscript letters indicate statistical differences between groups (P < 0.05). Repeated measures ANOVA was applied for statistical analysis.

**Supplementary Table** **4.** Proportion of Dissolved 3-week-old Plaque Biofilm during Exposure to Disinfecting Agents.

| Time (min) | 2% NaOCl^a^ | 6% NaOCl^b^ | 2% CHX^c^ |
| --- | --- | --- | --- |
| 2 | 0.24±0.06 | 0.54±0.06 | 0.07±0.03 |
| 4 | 0.23±0.07 | 0.58±0.07 | 0.04±0.05 |
| 6 | 0.37±0.07 | 0.65±0.06 | 0.09±0.05 |
| 8 | 0.45±0.06 | 0.68±0.05 | 0.05±0.05 |
| 10 | 0.53±0.05 | 0.71±0.05 | 0.15±0.09 |
| 12 | 0.58±0.06 | 0.76±0.04 | 0.04±0.06 |
| 14 | 0.58±0.06 | 0.82±0.03 | 0.06±0.15 |
| 16 | 0.57±0.07 | 0.83±0.03 | -0.03±0.09 |
| 18 | 0.56±0.07 | 0.85±0.02 | -0.08±0.12 |
| 20 | 0.63±0.06 | 0.86±0.02 | 0.02±0.08 |
| 22 | 0.61±0.06 | 0.83±0.03 | 0.09±0.10 |
| 24 | 0.61±0.06 | 0.82±0.03 | -0.04±0.14 |
| 26 | 0.70±0.06 | 0.84±0.03 | 0.17±0.13 |
| 28 | 0.72±0.07 | 0.86±0.02 | 0.13±0.09 |
| 30 | 0.76±0.06 | 0.85±0.03 | 0.16±0.12 |
| 32 | 0.75±0.06 | 0.86±0.03 | 0.07±0.09 |

Different superscript letters indicate statistical differences between groups (P < 0.05). Repeated measures ANOVA was applied for statistical analysis.

**Supplementary Table** **5.** Proportion of Killed Biofilm Microbial Cell Biovolume (PI-Stained Bacteria) in Residual 3-day-old Biofilm during Exposure to Disinfecting Agents.

| Time (min) | Sterile water^a^ | 2% NaOCl^b^ | 6% NaOCl^c^ | 2% CHX^b^ |
| --- | --- | --- | --- | --- |
| 2 | 0.05±0.01 | 0.28±0.04 | 0.85±0.02 | 0.20±0.01 |
| 4 | 0.04±0.01 | 0.29±0.04 | 0.95±0.01 | 0.27±0.02 |
| 6 | 0.04±0.01 | 0.33±0.05 | 0.97±0.01 | 0.45±0.02 |
| 8 | 0.04±0.01 | 0.38±0.04 | 0.94±0.01 | 0.40±0.02 |
| 10 | 0.04±0.01 | 0.48±0.07 | 0.93±0.01 | 0.40±0.04 |
| 12 | 0.04±0.02 | 0.51±0.11 | 0.96±0.01 | 0.54±0.02 |
| 14 | 0.05±0.01 | 0.48±0.11 | 0.89±0.02 | 0.52±0.06 |
| 16 | 0.04±0.02 | 0.52±0.12 | 0.84±0.03 | 0.56±0.02 |
| 18 | 0.04±0.02 | 0.53±0.12 | 0.79±0.03 | 0.57±0.02 |
| 20 | 0.04±0.01 | 0.55±0.12 | 0.78±0.06 | 0.66±0.02 |
| 22 | 0.04±0.02 | 0.56±0.12 | 0.74±0.10 | 0.66±0.02 |
| 24 | 0.04±0.02 | 0.56±0.12 | 0.72±0.07 | 0.58±0.07 |
| 26 | 0.04±0.02 | 0.71±0.04 | 0.69±0.09 | 0.69±0.06 |
| 28 | 0.04±0.02 | 0.73±0.08 | 0.76±0.06 | 0.71±0.02 |
| 30 | 0.04±0.02 | 0.65±0.07 | 0.65±0.08 | 0.65±0.07 |
| 32 | 0.04±0.02 | 0.75±0.04 | 0.64±0.10 | 0.73±0.12 |

Different superscript letters indicate statistical differences between groups (P < 0.05). Repeated measures ANOVA was applied for statistical analysis.

**Supplementary Table** **6**. Proportion of Dissolved and Killed Residual Bacteria (PI-Stained Bacteria) in 3-week-old Biofilm during Exposure to Disinfecting Agents.

| Time (min) | 2% NaOCl^a^ | 6% NaOCl^b^ | 2% CHX^c^ |
| --- | --- | --- | --- |
| 2 | 0.45±0.05 | 0.93±0.00 | 0.26±0.02 |
| 4 | 0.46±0.06 | 0.98±0.00 | 0.30±0.03 |
| 6 | 0.58±0.05 | 0.99±0.00 | 0.50±0.03 |
| 8 | 0.66±0.04 | 0.98±0.00 | 0.43±0.03 |
| 10 | 0.76±0.05 | 0.98±0.00 | 0.49±0.08 |
| 12 | 0.79±0.06 | 0.99±0.00 | 0.56±0.03 |
| 14 | 0.78±0.07 | 0.98±0.00 | 0.55±0.04 |
| 16 | 0.79±0.07 | 0.97±0.00 | 0.55±0.05 |
| 18 | 0.79±0.07 | 0.97±0.00 | 0.53±0.08 |
| 20 | 0.83±0.06 | 0.97±0.01 | 0.67±0.03 |
| 22 | 0.83±0.06 | 0.96±0.02 | 0.69±0.04 |
| 24 | 0.83±0.06 | 0.95±0.02 | 0.57±0.05 |
| 26 | 0.91±0.01 | 0.95±0.02 | 0.75±0.03 |
| 28 | 0.92±0.03 | 0.97±0.01 | 0.75±0.03 |
| 30 | 0.91±0.03 | 0.95±0.02 | 0.70±0.10 |
| 32 | 0.94±0.02 | 0.95±0.02 | 0.75±0.03 |

Different superscript letters indicate statistical differences between groups (P < 0.05). Repeated measures ANOVA was applied for statistical analysis.
